# ‘We want beer’: an analysis of online posts written about the alcohol ban during Qatar’s soccer world cup

**DOI:** 10.1093/alcalc/agaf059

**Published:** 2025-09-16

**Authors:** Erin Santamaria, Dan Anderson-Luxford, Zhen He, Emmanuel Kuntsche, Aiden Nibali, Jessica Ison, Nikita Potemkin, Benjamin Riordan

**Affiliations:** Centre for Alcohol Policy Research, NR1 Main Drive, 3084, La Trobe University, Melbourne, Australia; Centre for Alcohol Policy Research, NR1 Main Drive, 3084, La Trobe University, Melbourne, Australia; Computer Science and Information Technology, La Trobe University, Main Drive, 3084, Melbourne, Australia; Centre for Alcohol Policy Research, NR1 Main Drive, 3084, La Trobe University, Melbourne, Australia; Computer Science and Information Technology, La Trobe University, Main Drive, 3084, Melbourne, Australia; La Trobe Rural Health School, La Trobe University, Edwards Road, Flora Hill, VIC, 3552, Australia; Centre for Alcohol Policy Research, NR1 Main Drive, 3084, La Trobe University, Melbourne, Australia; Centre for Alcohol Policy Research, NR1 Main Drive, 3084, La Trobe University, Melbourne, Australia

**Keywords:** alcohol, sport, social media policy

## Abstract

**Introduction:**

The FIFA Men’s World Cup is one of the largest global sporting events, with alcohol playing a notable role, evident from major sponsor Anheuser-Busch. Indeed, Brazil altered policies to allow alcohol in the 2015 tournament stadiums. Qatar, the 2022 World Cup host, initially assured alcohol availability in stadiums but reversed this decision 2 days before the tournament began, sparking widespread online discussions about alcohol’s role in sports and policies. This study analyses the online discourse surrounding this alcohol ban.

**Methods:**

We collected Tweets from a week prior to the tournament to a week after, using keywords referencing the World Cup, alcohol, and the ban. After excluding retweets, 5252 independent posts were coded by stance on the ban and underlying rationale.

**Results:**

Anti-ban tweets dominated (55.1%; 15.9% pro-ban; 29% neutral). We identified five primary themes. Three opposing the alcohol ban: (i) Timing Backlash: Sudden implementation, (ii) The Power of Budweiser: Impact on sponsor brands, and (iii) A Troubled World Cup: Contribution to other issues, and two supporting it: (iv) Spectator Sobriety: Alcohol’s relevance in sport, and (v) Dry Stands, Safer Crowds: Potential to mitigate public disturbances.

**Discussions and conclusions:**

While just over half of Tweets opposed the alcohol ban, they focused on the ban's enactment and concerns over sponsor agreements rather than the absence of alcohol itself. Conversely, pro-ban tweets highlighted improved fan experience. Policymakers should consider how alcohol companies use social media to shape public opinion. Analysing online discourse can provide valuable insights for implementing and reviewing alcohol control strategies in sport.

## Teaser

**Short Summary:** This study examined X (Twitter) discourse on the 2022 FIFA Men’s World Cup alcohol ban in Qatar. Analysing 5252 tweets, 55.1% opposed the ban, primarily criticising timing and sponsor influence, while 15.9% supported it, citing improved fan experience. Findings underscore social media's role in public opinion and inform alcohol policy in sports.

## Introduction

From renditions of ‘Seven Nation Army’ to ‘Que Sera, Sera’, soccer fans are known for the elaborate and impressive chants they use to help support their team or criticize their opponents. The opening game of the 2022 Men’s soccer World Cup in Qatar, however, kicked off to the tone of something different, with Ecuadorian fans chanting ‘Queremos Cerveza,’ translating to ‘We want beer’. This sentiment was prompted by FIFA (Fédération Internationale de Football Association) and Qatar’s sudden announcement that alcohol would be banned within and around the tournament’s stadia, despite Budweiser being a major sponsor of the tournament. This shift in policy, announced just 2 days prior to the tournament's kick-off, marked a departure from FIFA’s previous stance on alcohol (Panja 2022), which had been an integral part of FIFA World Cup matches prior to 2022 (Dun and Rachdi 2023). The abrupt policy change sparked online discourse on the role of alcohol in sport, with many web-users voicing their opinions on the restrictive alcohol policy to be enforced in Qatar stadiums. The amount of public discourse around alcohol at the Qatar World Cup provides a key opportunity for researchers to analyse the discussions around the role of alcohol in major sporting events.

## Alcohol’s role in FIFA World Cup tournaments

Qatar is deeply influenced by the values of its official religion, Islam, which plays a significant role in shaping the country’s policies and social norms around alcohol sale and consumption (Al-Ansari et al. 2016). The country adheres to Islamic Sharia law in which alcohol is forbidden and thus is strongly regulated in public and commercialized settings. Most venues do not allow alcohol, and of the minority that do, purchasing restrictions and inflated alcohol prices are used as a deterrence (Michalak 2006). Since 1986, FIFA has retained Budweiser (a company of Anheuser Busch) as the official beer sponsor of its Men’s World Cup events with alcohol a very visible aspect of the events. Thus, it’s unsurprising that alcohol consumption during the tournament was a concern for Qatari nationals leading up to the staging of the 2022 World Cup (Al-Emadi et al. 2022). Nevertheless, Qatar successfully secured the winning bid for FIFA’s international tournament with an awareness of the established precedent set by previous FIFA World Cup’s regarding alcohol accessibility at matches.

Formerly, FIFA displayed a distinct unwillingness to accommodate host country’s restrictive alcohol policies at its international tournaments. For example, prior to the 2014 Men’s World Cup, many Brazilian states enforced legislation between 1996 and 2012 prohibiting alcohol sales and consumption by fans in soccer arenas as a preventative measure against incidents of spectator violence (de Almeida and Marchi Júnior 2020). Research on the aftermath of this alcohol policy revealed a decrease in violent police incidents and an increase in public stadium attendance (Brandão et al. 2020). After Brazil’s acceptance to host the 2014 competition, FIFA expressed its concern about Brazil’s alcohol and sport laws, pressuring the Brazilian government to hastily approve a specialized World Cup General Law in 2012 (Joseph 2014) designed specifically to favour FIFA’s sponsor, Budweiser, in promoting unrestricted beer sales (Jerabek et al. 2017). FIFA’s then General Secretary Jerome Valck stated, ‘Alcoholic drinks are part of the FIFA World Cup, so we’re going to have them… that’s something we won’t negotiate’ (Chappell 2012). This new legislation permitted alcohol in Brazil’s 2014 World Cup stadiums. FIFA’s involvement in Brazil’s national laws in favour of their own alcohol agenda and corporate aims displays both the power of FIFA, but also their belief that alcohol is of clear importance at sporting events.

### Alcohol and sport: a complex relationship

Research indicates a relationship between risky alcohol consumption at major sporting events and an increase in alcohol-related harms (Reichenheim et al. 2011, Miller et al. 2013). Risky drinking is defined as consuming five or more standard drinks in one session (National Health and Medical Research Council 2020). In many high-income countries, spectators at professional sporting events tend to engage in risky alcohol consumption that surpasses the levels seen in the general population (Kingsland et al. 2013). Such behaviours have been documented in studies across Australia, the UK, and Aotearoa New Zealand, with club members of community sporting leagues reporting heightened alcohol intake during matches compared to their usual consumption levels (Duff and Munro 2007, O'Brien et al. 2007, Rowland et al. 2012).

The ramifications of risky drinking, in general, extend to both individuals and others. Immediate risks for the individual include physical health problems, and a higher likelihood of accidents and injuries (Dawson et al. 2008, Kuntsche et al. 2017). Furthermore, the harm caused by others’ drinking can manifest as verbal, physical, or emotional abuse (Laslett et al. 2011, Seid et al. 2015, Lam et al. 2019). While heavy drinking itself does not directly cause violent behaviour, impaired judgment, and heightened reactivity due to acute alcohol intoxication can contribute to violent behaviour (Attwood and Munafò 2014). Notably, women are more likely than men to report feelings of fear and instances of harassment and other negative impacts in public and private settings due to the drinking behaviour of others (Stanesby et al. 2018, Lam et al. 2019). Although the harms extend beyond the individual to impact wider public systems. In Australia, major events like the Australian Football League Grand Final and the Melbourne Cup have seen a surge in cases of acute alcohol intoxication requiring medical aid (Lloyd et al. 2013), with excessive alcohol consumption on major sporting event days increasing hospital emergency department visits, which could pose a strain on public health services (Hagan et al. 2024). In the instance of justice departments, studies suggest a link between heavy alcohol use and disturbances among sport spectators near professional stadiums (Durbeej et al. 2016, Klick and MacDonald 2021). For instance, a study of 49 US law enforcement agencies found over 75% reported alcohol-related violence incidents occurring around stadiums involved stadium spectators (Lenk et al. 2009). Risky alcohol consumption at sporting events can lead to greater instances of harm for individuals and strains public services, of which strong alcohol polices within stadia have the potential to mitigate.

Gauging public opinion on alcohol policies is central to understanding broader societal perceptions of alcohol, and in turn, public willingness to support health-orientated alcohol policies. On a broader scale, previous studies have demonstrated that support for alcohol policies (e.g. limiting availability, excise taxation, marketing restrictions) is associated with a greater awareness of the risks linked with alcohol use (Bates et al. 2018, Weerasinghe et al. 2020). This was true for Australian elite sport spectators whose support for restrictive policies against unhealthy commodities like alcohol was linked to a greater understanding of the harm caused by these products (Boelsen-Robinson et al. 2022). In addition, the introduction of alcohol control policies shapes public attitudes (Wallin and Andréasson 2005, Tobin et al. 2011). An example of this was observed following the introduction of random breath testing in Australia, where a survey on Australian drivers’ attitudes and behaviours towards the policy revealed an increase in social disapproval and stigmatization of drink driving. This, alongside the deterrent effect, significantly impacted the prevalence of drink driving behaviour (Job et al. 1997). Thus, by measuring public attitudes greater insight can be gained about broader attitudes to alcohol, as well as the impacts of policy on subsequent alcohol use behaviour.

Most of the studies to-date that have considered public support for alcohol in sport have focused on attitudes towards alcohol sponsorship (Boelsen-Robinson et al. 2022, McDaniel and Mason 1999, Surujlal and Dhurup 2009). In a cross-country survey conducted by Dekker et al. (2020), examining public support for various alcohol policies, it was found that, on average across the surveyed nations, 51% of participants favoured prohibiting alcohol sponsorship during televised sports events, 56% at sporting venues, 53% within professional settings, and 55% within community sporting clubs. However, there were significant cross-country differences with the largest support for bans in China and India, and the lowest support in the United States. Furthermore, Australian elite sports fans favour policies that restrict alcohol sponsorship visibility over complete removal due to concerns for team revenue (Boelsen-Robinson et al. 2022).

Several studies have specifically examined support for alcohol consumption in professional sports stadia. In the UK, alcohol restrictions are in place at soccer stadiums, with rules varying between regions. In England and Wales, alcohol use is prohibited ‘within sight of the pitch,’ while in Scotland, consumption is limited to designated hospitality areas away from the playing area (Martin et al. 2022). Martin and colleagues conducted focus groups with UK soccer fans, finding that the majority favoured a review of these laws. Another study of UK soccer fans (n = 1750) reported that 74% accepted drinking at stadiums, 76% believed alcohol should be available, and 35.6% thought it should be easily accessible (Purves et al. 2022). A Danish study that surveyed 5133 participants in 2011 found that 34.4% of participants supported a ban on the consumption and sale of alcohol at sporting events (Elmeland and Villumsen 2013). Conversely, a cross-sectional study of Swedish football fans found that only 26% supported the sale of alcohol at football events (Skoglund et al. 2017). Notably, many of these studies utilized traditional survey methods or topic-guided small group discussions, both of which entail contrived situations prone to response bias and can have resource-intensive requirements (Coughlan et al. 2009) among other limitations. While these findings highlight crucial attitudes towards alcohol policies in sport settings, there stands a need for more varied research methods to gauge public opinions on alcohol and sport in a less artificial manner.

Social media platforms offer a unique insight into public attitudes and have the potential to overcome some of the limitations of traditional survey methodologies (Dong and Lian 2021). Social media platforms, such as X (formerly Twitter) or Reddit, offer access to a large swath of publicly accessible information on public opinion. Some research has explored how social media can be used to examine public reactions to health-related announcements, such as the COVID-19 vaccinations (Hoque et al. 2022, Cho et al. 2024) or support for cannabis legalisation (Riordan et al. 2021). In contrast, even if representatively sampled, most survey methodologies rely on a comparatively limited sample of participants (Dong and Lian 2021). Similarly, as Tobin et al. (2011) aspects of survey design, such as the framing, order and wording of questions can risk the validity and reliability of such approaches. Finally, the large-scale assessment of public sentiment can be conducted in close to real-time, and thus, provides an efficient and less resource intensive solution than survey methodologies (Heiervang and Goodman 2011).

### The current study

The abrupt alcohol ban at the Qatar World Cup, announced only 48 hours before tournament commencement, provides a unique opportunity to investigate public sentiment around the role of alcohol at sporting events globally. Considering this, we used a thematic analysis of all posts on Twitter (now X) in the week prior to and proceeding the tournament to determine the proportion of posts that express support for the ban (i.e. ‘pro-ban’), take a neutral stance, or are against the ban (i.e. ‘anti-ban’). In addition, we explore the potential reasons for the pro- and anti-attitudes towards the ban. Considering the potential for alcohol bans at sporting events to significantly reduce alcohol-related harms (Lenk et al. 2009), this study will provide critical understandings of both current support for and possible barriers to alcohol control policies at international sporting events, and in turn, may provide support for the future development of such strategy.

## Methods

### Data collection

To collect posts, we used the R package academictwitteR to interact with Twitter’s academic Application Programming Interface (API), which allowed us to use search terms and to collect publicly available Tweets. For our search, we specified that posts should be original posts (excluding reTweets), written in English, and written between 3/11/2022 and 24/12/2022 (7 days prior to the start of the 2022 World Cup and seven days after the World Cup to account for both the official announcement and the preceding public discourse). We included the specific keywords that referenced ‘Qatar,’ ‘World Cup,’ ‘FIFA,’ ‘alcohol,’ ‘ban,’ ‘beer,’ ‘wine,’ ‘champagne,’ and ‘booze.’ This search was conducted in December 2022 and yielded 5252 total Tweets within the above selection criteria for analysis. The data were transported into Excel for analysis.

### Data coding and analysis

We used a reflexive thematic analysis approach (Braun and Clarke 2022). We chose this approach as it allowed for multiple steps of data analysis while approaching analysis through reflection rather than finding a ‘truth’ in the data. For step one, ES and NP created columns in Excel for pro-, anti, or neutral. They then coded the Tweets into pro-, anti-, or neutral the alcohol ban. A third annotator BR then read through and resolved any disagreements or flagged some posts for further discussion to be resolved as a team. We resolved any discrepancies to create a dataset with 100% agreement to test the accuracy of a machine learning algorithm for a separate project. We defined posts as pro if they showed agreement with or approval of the alcohol ban and its repercussions, anti if they expressed dislike of or disagreement with the alcohol ban and its repercussions, and neutral if they were plain factual statements on the events that occurred (no emotive language). See Table 1 for examples of pro, anti, and neutral posts.

**Table 1:** Twitter posts for stance on the alcohol ban at the Qatar World Cup stadiums.

Pro-ban	Anti-ban	Neutral ban
“Why does watching soccer require alcohol? You can enjoy it without.”	“Reports are in that Qatar is banning beer at the World Cup in a dramatic U-turn two days before the tournament.”	“Qatar plans to ban alcohol at the World Cup stadiums”
“They are definitely making the right decision banning alcohol!”	“Fans who have shelled out thousands travelling to Qatar demand refunds after shock beer ban.”	“World Cup officials ban beer from stadiums two days before the tournament begins”
“Women enjoy a hassle-free experience after the alcohol ban within Qatar stadiums”	“Qatar and FIFA beer ban is a very expensive headache for Budweiser with all that beer.”	“FIFA announces a ban on beer at World Cup stadiums”
“This World Cup has shown the world that sport and booze should be two separate things.”	“Soccer fans are angry after being faced with a ban on beer.”	“FIFA announces Bud Light will be available within stadiums after the alcohol ban.”

For step two, after the posts were categorized as either pro-, anti-, or neutral, the two annotators read the entire data set again to conduct a second round of coding. Each post was coded for the reasons it was in support of or opposed to the alcohol ban. Employing inductive coding, each annotator independently created a list of ‘why’ codes emerging from their initial data analysis when coding for pro-, anti-, or neutral stance. The two annotators discussed similarities and differences in their independently identified codes and synthesized these into a comprehensive master table of ‘why’ codes. This table was used to code reasons to be in support of or opposed to the alcohol ban for all pro- and anti-ban identified posts, although an ‘other’ code was included to incorporate reasons outside of the identified table where needed. For the third step, [redacted for review] met to discuss the themes they had identified in the ‘why’ codes. Together they generated five primary themes. For the final step, they sorted the codes into the themes through reading each ‘why’ post. They reflected and discussed the themes, adjusting as they coded. Furthermore, while the primary approach was a reflexive thematic analysis, we included select content analysis to quantify the prevalence of specific themes and provide additional context to support our interpretations.

### Positionality

The research team is inter-disciplinary, working across alcohol policy research, computer science, gender-based violence research, and data science with a commitment to research that is ethical and intersectional. The team members who coded the data are ES who holds an Honours in psychology, NP who holds a PhD in Neuroscience and BR is a research fellow who holds a PhD in Psychology. Throughout, they reflected on their assumptions and biases coding and discussed these with the wider team.

### Ethical considerations

While extracting online discourse provides the opportunity to analyse expansive data sets, there are concerns regarding poster anonymity as it is not possible to seek their consent for use of their data. In this project, we were guided by guidelines from other researchers on how to collect and analyse social media data (Colditz et al. 2018, O’Callaghan and Douglas 2021). To protect participants, we opted for a broad overview of the posts, rather than focusing on specific posts. Additionally, when we did report quotes, we used principles of Markham’s (2006) ethical fabrication which involved changing keywords (e.g. changing ‘Qatar World Cup bans alcohol in last-ditch U-turn’ to ‘Alcohol has been abruptly banned at Qatar World Cup stadiums’) to prevent back-searching on the internet, thus masking poster identity. Finally, we chose not to make our data open access, as we believe doing so could risk user identification and raise ethical concerns, despite the potential benefits of data sharing.

## Results

**Figure 1:**
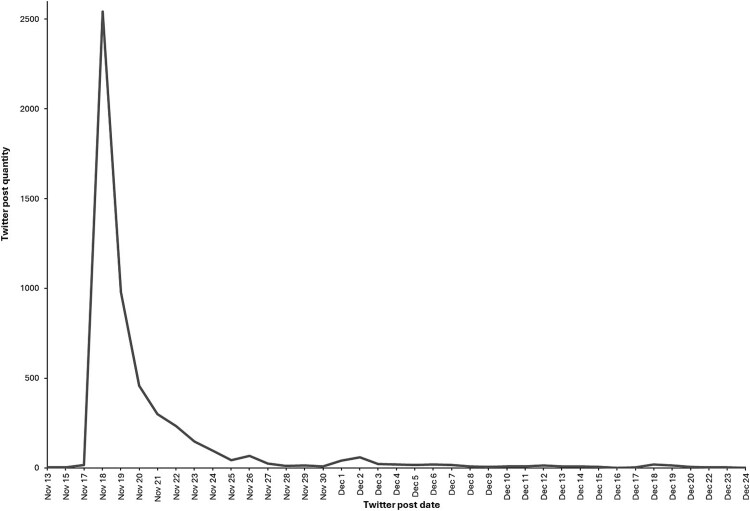
Number of Twitter posts in relation to the Qatar World Cup alcohol ban posted in 2022.

From the 5252 posts, 835 (15.9%) were pro, 2895 (55.1%) were anti, and 1522 (29%) were neutral. As seen in Fig. 1, most of the posts were written immediately after the ban announcement.

Five primary themes were developed from the analysis of Tweets which reflected the online posters’ reasoning for their stance on the sudden alcohol policy implementation, three related to posts opposing the alcohol ban (Timing Backlash, The Power of Budweiser, and A Troubled World Cup) and two related to posts in support (Spectator Sobriety, and Dry Stands, Safer Crowds).

### Timing backlash: criticisms on the ban’s sudden implementation

A common sentiment expressed in tweets opposing the alcohol ban revolves around the dissatisfaction with the implementation timeline set by Qatar and FIFA, which provided 48 hours’ notice. Many tweets shared or retweeted newspaper articles that highlighted the timing negatively, referring to it as a ‘last-minute decision,’ a ‘shocking U-turn,’ or an ‘abrupt ban on beer.’

Some tweets highlighted the implications of the ban’s timing on international travellers to the Qatar World Cup who had made plans and arrived with the expectation of being able to consume alcohol and celebrate, with reshared headlines such as ‘Travelling fans demand refund’ and posters commenting ‘Shame on FIFA. Fans have spent so much traveling to World Cup for Qatar to pull this last-minute ban.’

Additionally, some tweets specifically mentioned England and Wales fans, implying that they would be deeply disappointed and enraged by the sudden prohibition due to their customary binge-drinking behaviour during soccer matches: ‘Wales fans are expressing anger and frustration over the unexpected alcohol ban in stadiums.’

### The power of Budweiser: impact on sponsor brands

Budweiser, and its parent company Anheuser-Busch, emerged as a focal point in discussions surrounding the alcohol ban, as referenced in 17.7% of the tweets analysed (n = 930) using either the term ‘Budweiser,’ ‘Bud,’ ‘Anheuser Busch,’ or ‘InBev.’ Of these references, 678 (72.9%) were anti-ban, 59 (6.3%) were pro-ban, and 193 (20.8%) were neutral.

Many Twitter posters expressed outrage over the ban, speculating on its potential adverse effects on Budweiser’s ongoing sponsorship deal with the FIFA World Cup. Many highlighted the significant financial investment Budweiser made to sponsor the international tournament, which faced uncertainty following the ban’s announcement: ‘Shock beer ban despite Budweiser’s multi-million-dollar sponsorship deal.’ Some posters called for Budweiser to take legal action against FIFA for jeopardizing their beer sales: ‘I hope Budweiser sues the crap out of FIFA and Qatar.’

Budweiser was often depicted as a victim and praised for its handling of the situation in contrast to FIFA and Qatar. Several re-tweeted headlines emphasized how the alcohol company was ‘taken by surprise’ with the predicament: ‘A blow for Budweiser,’ ‘Bud left with excess beer on its hands,’ and ‘Budweiser’s World Cup strategy tempered but not shattered.’ Twitter posters praised Budweiser for its adept handling of the alcohol ban, both upon its announcement and in the ensuing aftermath. Budweiser’s approach included an initial light-hearted ‘this is awkward’ tweet, swiftly followed by the announcement that any unsold Budweiser beer would be generously donated to the winning team’s country: ‘Budweiser’s brilliant response to Qatar’s beer ban,’ ‘Making the best of a bad situation’ and ‘Kudos to Budweiser’s clever marketing scheme, with an innovative solution.’

### A troubled world cup: the ban’s contribution to other issues

Twitter posters viewed the alcohol ban as another unwelcome imposition amid an environment of other contentious issues surrounding the event. Some lumped the ban together with concerns about human rights violations, labour conditions, and restrictive laws concerning the LGBTQ+ community, portraying it as yet another negative aspect of the tournament: ‘Killjoy Qatar with too many rules for alcohol, women and LGBTQ community.’ Quite often posts from this theme linked closely with anti-Qatar sentiment as people expressed their dislike of the Qatar World Cup while expressing an anti-ban stance: ‘A whole mess in Qatar including the sudden alcohol ban, paying fans to attend, and more…#humanrights,’ ‘Now a last-minute alcohol ban?! They should not have been given the World Cup. Boycott Qatar!’

### Spectator sobriety: alcohol’s relevance in sport

The prevailing sentiment in supportive Tweets regarding the alcohol ban questioned the necessity of alcohol at sporting events: ‘The alcohol ban has demonstrated that organising a major sporting event without alcohol is feasible and embraced.’ Retweets of newspaper headlines highlighted the beer ban’s negligible impact on the international soccer tournament’s ambiance, with headlines like ‘Despite beer ban, World Cup revellers embrace the festive spirit.’ Posters conveyed their indifference toward alcohol or the notion that its absence did not detract from the enjoyment of attending matches: ‘You can still have a great time with or without alcohol,’ ‘While alcohol and soccer often go hand in hand, Qatar has proven otherwise,’ and ‘A step in the right direction away from the toxic combination of alcohol and sport.’

Several Tweets cited FIFA president Gianni Infantino’s remarks, emphasizing the idea that World Cup attendees could ‘survive without consuming alcohol for three hours a day’ and that the ban amounted to little more than a ‘minor inconvenience.’ Additionally, Twitter posters mocked other poster’s complaints against the alcohol ban: ‘people are acting like beer is a human right,’ ‘Well boo hoo to them, there are more important things then getting pissed at a football match,’ and ‘… just wait to be back in America to drink maybe? Show some respect.’

### Dry stands, safer crowds: a ban’s potential to mitigate public disturbances

Twitter posters voiced support for the alcohol ban citing its possible impact on reducing hooliganism and public disturbances, ensuring a safer crowd experience. Many highlighted the correlation between alcohol consumption and instances of rowdy behaviour: ‘Scenes of England and Wales supporters brawling in Tenerife is the reason why Qatar is banning alcohol,’ ‘UK soccer would be a lot less hostile without the drunken violence,’ and ‘With alcohol there is chaos, and in its absence, there is peace, decency and order.’ Other posters enthusiastically supported the idea of implementing an alcohol ban at sporting events after experiencing an alcohol free 2022 World Cup, advocating for their respective countries to adopt similar measures ‘The UK should impose this and save the police controlling hooligans and drunkards,’ and ‘Having witnessed the positive effects of an alcohol-free World Cup, it’s time for widespread adoption!’

Furthermore, there was discourse about the alcohol ban’s positive impact on women’s safety within the spectator crowds. Many asserted that women felt more secure without the presence of ‘drunken men harassing and making women uncomfortable.’ Following the World Cup, Twitter users echoed these sentiments, noting there was ‘less sexism with wolf whistles and catcalling,’ and with re-tweeted headlines such as ‘Women enjoying a hassle-free World Cup.’

## Discussion

The online discourse surrounding the sudden alcohol ban at the Qatar 2022 FIFA World Cup offered a unique and organic opportunity to gauge public opinion on alcohol’s role at international sporting events employing a method largely unused in this research area. The assessment of online Twitter posts revealed an overall negative sentiment towards the alcohol ban, which aligns with previous research findings that show public support for the sale and consumption of alcohol in sports stadiums (Elmeland and Villumsen 2013, Martin et al. 2022, Purves et al. 2022). Interestingly though, these anti-ban posts centred around the timing of the ban’s announcement and implementation, and its impact to sponsors rather than the prohibition of alcohol itself. This finding suggests that public opinion towards an alcohol ban at international sport tournaments may be influenced by considerations beyond the mere presence or absence of alcohol, and values focus on the impact on commercial partnerships and the overall experience for fans.

Concerns about supporting alcohol prohibition policies at professional international events are heightened by the potential impact on existing sport and alcohol sponsorship agreements. One notable reason for opposing the alcohol ban at the Qatar World Cup was concern for Budweiser’s established sponsorship with FIFA, which was greater than the concern for an already troubled World Cup. Additionally, the timing of the ban’s announcement and enforcement, only 48 hours before the first match, intensified the outrage in support of Budweiser and its jeopardized multi-million-dollar deal. Indeed, of the posts that mentioned Budweiser, the majority were anti the ban and showed a degree of support for the alcohol company. These findings differ from previous research suggesting support for the banning of alcohol sponsorships in sports stadiums (Surujlal and Dhurup 2009, Dekker et al. 2020). While concern for impacts to revenue is shown to impact support for restrictive alcohol policies in sport (Boelsen-Robinson et al. 2022), this centred around team revenue rather than that of alcohol companies. In the instance of international sport tournaments, where alcohol companies like Budweiser sponsor the tournament as a whole instead of individual teams, concerns for revenue may instead fall to the new ‘victim’ of stadium alcohol policies, the alcohol companies, particularly where a sponsorship deal is established and active. Our findings suggest consideration of negative sponsor impacts due to prohibitive alcohol sport policies are likely greater than previous research has suggested, particularly on the scale of international tournaments.

When public interests align with those of alcohol companies, advocating for public health policies becomes notably challenging. Our findings indicate that Twitter posters were against the sudden alcohol ban due to concerns for how it would impact Budweiser’s ability to sell beer within stadia, more so than their concern for simply accessing beer in general. A World Health Organization brief from 2021 highlighting conflicts of interest regarding alcohol policies, revealed that companies engaged in producing or selling unhealthy commodities, such as alcohol, actively impeded the design, implementation, and evaluation of control policies worldwide (World Health Organization 2022). Proposed societal-level measures to address these issues included exposing negative industry behaviour and collaborating with civil society to enhance industry accountability and practices. Interestingly, our data shows that Budweiser received largely positive, supportive posts and may have even received a degree of promotion through reference to them in just under a fifth of analysed posts, posing a potential barrier to such measure implementation. This brings into question how alcohol companies could and are using social media platforms to garner sympathy and support for them in the wake of alcohol policy design and review.

Regarding the posts in support of the ban, Twitter posters expressed their agreement with alcohol-free stadiums and the subsequent positive impact to fan experience the alcohol ban had. While posters referenced this in relation to decreased hooliganism, notably, it was emphasized that female attendees experienced less alcohol-related harms in the form of harassment by others within stadia. This finding may suggest women experience greater harassment than men in sport stadiums when alcohol is involved. While research indicates women generally report more harm from known others’ drinking than unknown (Seid et al. 2015), alcohol-related disturbances in major sport stadiums are likely influenced by stadiums-specific factors. These include mixed supporter proximity, rivalries, and championship stress, among others, which could amplify harms from unknown others’ drinking than normal (Ostrowsky 2018). Hence, when crafting or revising alcohol policies in sports, like those examined in the UK (Martin et al. 2022), it is imperative to consider the differing experiences of female attendees, who may be disproportionately affected by alcohol-related harassment within stadiums. Continued research and monitoring of alcohol-related disturbances in international sport stadiums, both through official reports and social media, could inform conception and promotion of evidence-based alcohol policies that prioritize fan safety and enjoyment.

Lastly, this study suggests that social media platforms offer a broader and less contrived avenue for assessing public opinions on health-related alcohol policies compared to traditional survey methods commonly used in this research area. Alcohol and sport are consistent topics of discussion in media globally, with recent topics around policies including the surprise alcohol-free trial for certain seated zones at Twickenham stadium in the UK (Morgan 2024), and the decision to maintain alcohol prohibition policies within French sports arenas at the 2024 Olympics (Salvador 2023). There is ongoing debate about the ethics of using social media data without user consent (Nicholas et al. 2020). While obtaining informed consent is often impractical for large-scale datasets, such as those based on Twitter or Instagram hashtags, some argue that users implicitly consent through platform terms and the public nature of posts (Moreno et al. 2013, Beninger et al. 2014). Nonetheless, this research was conducted with care, and future work should continue to engage with the evolving ethical landscape of using social media data for public opinion research. Analysing online responses to such news from social media posters could allow policy makers to determine public opinion and perceived success rate of trial policies in real time and with larger scopes than targeted surveys have previously allowed.

### Limitations

While utilising social media for research is gaining popularity for its expansive reach and capacity to analyse large datasets, pinpointing the geographical location of Twitter posters poses challenges unless explicitly provided by the posters, a rarity in our sample. Consequently, we cannot draw conclusions about public opinion on alcohol policies in specific locations, nor were we able to use user demographics to determine if any of the collected comments were bot-generated. Using bots may be a particularly attractive method for lobbying groups to shape public discourse and future research should consider methods to detect bot-like activity [see (Lopez-Joya et al. 2023) for a review of strategies]. This may be particularly important as Large Language Models like Chat-GPT make it easier to automate content. A limitation of Twitter, and social media more broadly, is that it caters to individuals with internet access, electronic devices, and a desire to express themselves online, thus excluding those without such means. And as we excluded posts not written in English, we may have missed important data from posts in other languages.

Furthermore, since data collection, Twitter (now X) has undergone significant changes, including removing the free AcademicAPI that we used to collect this data. Although researchers can now pay to use Twitter/X data, there has been a decline in the use of the site. Additionally, research has shown that industries such as ultra-processed food use platforms like Twitter to shape policy discourse and public opinion (Hunt 2021). While this study did not examine potential astro-turfing or lobbying activity by alcohol companies or affiliated stakeholders, this remains an important avenue for future research. The current analysis focused on capturing the overall sentiment expressed publicly on Twitter/X, content that would have been visible to other users and part of the broader online discourse.

Finally, Qatar’s Islamic culture and religious beliefs concerning public alcohol consumption influenced the decision to prohibit alcoholic beverages in their World Cup stadiums. We recognize that Islamophobia or issues with Qatar hosting the Men’s Soccer World Cup likely influenced some Twitter users’ opposition to the alcohol ban although it was beyond the scope of the current paper to appropriately address this aspect. Even so, future research should explore how attitudes towards own and other’s religion impact support for and adherence to alcohol control policies at major international sport tournaments.

## Conclusions and implications

Examining online discourse prompted by real-world events is a novel methodological approach in assessing public support for and possible barriers to alcohol control policies at major international sporting events, as well as understanding alcohol’s role in sport. Based on our analysis of Twitter posts regarding the sudden alcohol ban at the Qatar 2022 men’s FIFA World Cup, our findings reveal that public opposition to such policies is more significantly shaped by concerns regarding negative impacts to existing alcohol sponsor agreements than previous research has suggested. Additionally, public sentiments toward enhancing fan experience and ensuring safer environments, particularly for women, contribute to greater support for restrictive alcohol policies within stadiums, highlighting that the mere presence or absence of alcohol is not the sole determinant of public opinion. In developing new alcohol control strategies for international sporting events, policymakers should prioritize understanding how alcohol companies leverage social media platforms to shape public support. Additionally, analyses of public social media posts in future research offers an organic opportunity to continue informing policy makers in the implementation and review strategies targeting risky alcohol consumption in major sport stadiums.

## Ethics number

HEC22335.

## Data Availability

Due to ethical considerations the data associated with this project are not available on request.
